# Study of Local Hydrodynamic Environment in Cell-Substrate Adhesion Using Side-View μPIV Technology

**DOI:** 10.1371/journal.pone.0030721

**Published:** 2012-02-17

**Authors:** Yi Fu, Robert Kunz, Jianhua Wu, Cheng Dong

**Affiliations:** 1 School of Bioscience and Bioengineering, South China University of Technology, Guangzhou, China; 2 Bioengineering Department, Pennsylvania State University, University Park, Pennsylvania, United States of America; 3 Applied Research Laboratory, Pennsylvania State University, University Park, Pennsylvania, United States of America; University of Arizona, United States of America

## Abstract

Tumor cell adhesion to the endothelium under shear flow conditions is a critical step that results in circulation-mediated tumor metastasis. This study presents experimental and computational techniques for studying the local hydrodynamic environment around adherent cells and how local shear conditions affect cell-cell interactions on the endothelium in tumor cell adhesion. To study the local hydrodynamic profile around heterotypic adherent cells, a side-view flow chamber assay coupled with micro particle imaging velocimetry (μPIV) technique was developed, where interactions between leukocytes and tumor cells in the near-endothelial wall region and the local shear flow environment were characterized. Computational fluid dynamics (CFD) simulations were also used to obtain quantitative flow properties around those adherent cells. Results showed that cell dimension and relative cell-cell positions had strong influence on local shear rates. The velocity profile above leukocytes and tumor cells displayed very different patterns. Larger cell deformations led to less disturbance to the flow. Local shear rates above smaller cells were observed to be more affected by relative positions between two cells.

## Introduction

Cancer has become one of the main causes of death for human beings in the world [1]. Although different research fields are focused on cancer therapy, many challenges remain to be conquered [2]–[11]. Tumor metastasis is a complex process with multiple steps, (e.g., heterotypic cell adhesion and cell extravasation from the blood post-capillary vessel, and soluble protein factors involved during the process.) Therefore, it is of great interest to understand the mechanisms behind metastasis and develop better therapeutic targets [5], [12]–[17]. Melanoma, one type of tumor cells (TCs), is responsible for skin cancer which widely threatens human lives [18], [19]. Previous studies have indicated that human melanoma cells actively recruit leukocytes, especially neutrophils (PMNs), to adhere to the vascular endothelial cells (ECs) under high shear flow conditions, because the receptor-ligand bonds between melanoma cells and ECs were insufficient to enable initial capture of melanoma cells on vessel walls [20]–[26]. Moreover, plasma proteins, like fibrinogen or fibrin, could also act as a bridge for TC binding to EC [27]. The mechanisms of PMN facilitated TC-EC adhesion and subsequential extravasation, first developed and studied in our lab, has indicated that PMN plays an important role in melanoma extravasation and metastasis [21], [23]–[25].

Micro-particle imaging velocimetry (μPIV), first developed from the conventional PIV [28], [29], has been widely applied to microfluidic studies in recent years [30], [31], and has enabled successful velocity measurements in a near-wall region of micro channels [32], [33]. The flow microenvironment and velocity profile in local regions of a blood vessel is important since important biological phenomena are affected by local flow conditions (e.g., flow-enhanced cell adhesion, and, flow-induced cell deformation [34]–[37].) μPIV combined with other techniques, such as confocal microscopy and side-view imaging, has recently been applied for in *vivo* and in *vitro* studies [38], [39]. Employing μPIV to study the flow field in the vicinity of ECs and shear effects on the ECs has been conducted for several years [40]–[42]. For example, Pommer *et al.* measured the shear stress distribution around a single adherent red blood cell in a microchannel with μPIV [43]; Lee *et al.* applied μPIV to blood flow measurements in extraembryonic blood vessels of chick embryos [44], and Poelma *et al.* recently developed a methodology based on μPIV to determine the wall shear stress in vivo in the vitelline network of a chicken embryos [45], [46]. Moreover, the properties of blood flow around red blood cells, as well as the disturbance of blood flow caused by thrombosis at different layers in an in vitro flow chamber were studied by Sugii, Lima and *et al.* [38], [47]. Most recently, Jordan Leyton-Mange *et al.* successfully combined μPIV with a side-view imaging technique to study the local hydrodynamic environment around a single Jurkat cell adhered to ECs under different flow conditions, and the results indicated that local shear rates above an adherent cell are significantly different from that of upstream near-wall regions and are affected by deformability of the cell [39].

Computational fluid dynamics (CFD) has been widely applied to study cells in blood vessels. μPIV has served as a useful validation tool for these studies [48]. Dong *et al.* established a 2-D model to study the deformation of an adherent leukocyte under different shear conditions based on side-view experimental images [37], [49]. Multi-phase CFD was recently applied to analyze monocyte adhesion by Lyczkowski *et al.* [50]. Pappu *et al.* and Jadhav *et al.* developed a 3-D model for leukocyte adhesion and rolling in a shear flow [51], [52]. Jadhav *et al.* successfully modeled the collision and interaction between two deformable cells to study the contact area between homogeneous and heterogeneous cells [53]. Munn *et al.* studied interactions between white blood cells and red blood cells in a near wall region under flow conditions [54]. Pawar *et al.* investigated cell rolling at three different scale levels including cell deformation (mesoscale), microvillus deformability (microscale) and receptor-ligand binding kinetics (nanoscale) by coupling immersed boundary method, Hookean spring model and Monte Carlo method [55]. Hoskins *et al.* developed a CFD model coupled with biochemistry and adhesion kinetics to study the process how a TC interacts with an adherent PMN on the EC [56].

Although the mechanism of PMN-facilitated TC-EC adhesion (directly or indirectly) has been actively studied, the modulation of local hydrodynamic environment by the process is not well understood. Nevertheless, the local fluid dynamics is strongly coupled to cell deformation and adhesion, and it is therefore critical to the understanding of the physical processes of these systems. For example, an interesting phenomenon observed in previous flow chamber experiments is that moving TCs tend to be captured by an adherent PMN on its downstream side, not upstream or lateral sides. We are interested in how the fluid dynamics couples with the cell structural mechanics and biochemistry in these systems in such a way as to give rise to such observations. The specific purpose of this paper is to use combined side-view μPIV technique and CFD to establish a methodology for studying the local fluid dynamics around a pair of PMN and melanoma cells with different relative PMN-to-melanoma positions under different flow conditions. Although other studies have measured the velocity profiles around homoeotypic cells [41], [43], these were measured either two-dimensionally (top-view) [43] or using 3-D reconstruction [57]. Here, we have improved our experiment setup and data analysis process for side-view μPIV and successfully measured the velocity profile above an adherent PMN, the size of which is small (8 µm) compared with previous studies (22 µm) [39]. Moreover, we have perfused two types of cells into the side-view chamber at the same time and obtained velocity profiles above two adherent cells at five PMN-to-TC position states reflecting the process of PMN-mediated TC adhesion.

## Materials and Methods

### Cell culture and preparation

The study was specifically approved by The Pennsylvania State University Institutional Review Board (IRB), and written consents were obtained from all participants. Fresh blood was drawn from healthy adult volunteers following The Pennsylvania State University IRB-approved protocols (No. 19311) and PMNs were separated according to previously published protocols [21] by using Ficoll-Hypaque density gradient Histopaque-1077 and Histopaque-1119 (Sigma; St. Louis, MO). After collection, PMNs were resuspended at a concentration of 1×10^6^ cells ml^−1^ in Dulbecco's phosphate-buffered saline (DPBS) containing 0.1% HSA (Calbiochem; La Jolla, CA) and stored at 4°C up to 4 hours until experiment.

Lu1205 (provided by Dr. Gavin Robertson, Penn State University Hershey Medical Center, Hershey, PA) melanoma cells were maintained in suspension of DMEM/F12 (Dulbecco's Modified Eagle Medium Nutrient Mixture F12, GIBCO; Carlsbad, CA) containing 10% FBS (BioSource; Carlsbad, CA) and put in a 37°C tissue culture incubator supplemented with 5% CO_2_. When cells became sub-confluent in the tissue culture dish, they were detached by applying 0.05% typsin/EDTA (Invitrogen; Carlsbad, CA) and washed twice with fresh culture medium. The cells were then mixed with fresh culture medium at 37°C for 1 hour to recover, after which the cells were centrifuged and resuspended at a concentration of 1×10^6^ cells ml^−1^ in DMEM/F12 containing 1% BSA (Sigma; St. Louis, MO) for experiment.

### Coupled side-view μPIV setup

The side-view μPIV flow system used in this research was previously developed by Cao *et al.* [58]. Briefly, microslides Vitrotubes™ #5005 and Vitrocells™ #8270 (Vitrocom; Mountain Lakes, NJ) were washed in double distilled water and ethanol. Microslides were stored in a clean environment after sterilization until being used. Prior to an experiment, microslides #5005 with an outer dimension of 700 µm (width)×150 µm (height) were coated with 20 µg ml^−1^ Fibronectin (BD; Sparks, MD) at 37°C for 2∼4 hours and then treated with DPBS containing 1% BSA at room temperature for 1 hour to eliminate nonspecific binding.

A coated microslide #5005 was then inserted into a larger microslide #8270 (inner dimension 700 µm (width)×700 µm (height)) to form a flow channel; two needles with tubing connected were inserted in both sides of the flow channel to hold the smaller microslide stably on the bottom of the channel. Super glue was added on each side to seal the chamber. The final dimension of flow field was 700 µm (width)×550 µm (height) with an aspect ratio of 1.27. The flow within the channel is fully developed where PIV observations are made since the Reynolds number is very low. Cao *et al.* [58] introduced a correction factor applied to analytical (parabolic) 2D channel velocity profiles, to estimate wall shear stress distributions across the width of the flow channel with respect to a chosen finite aspect ratio (Eq. 1).

**Figure 1:**
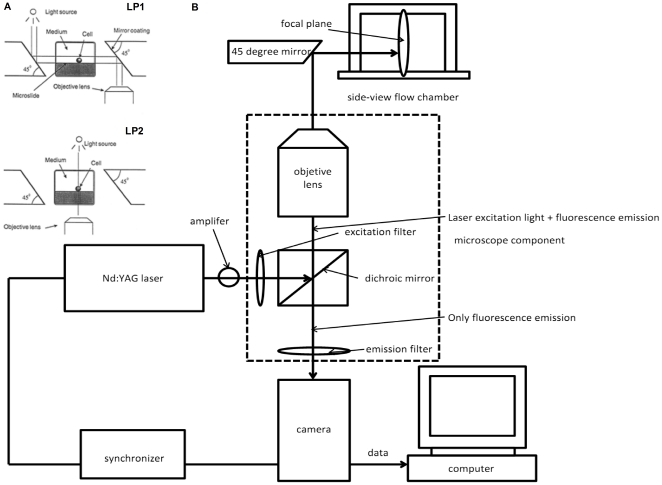
Schematic diagram of a coupled side-view μPIV system (not to scale). A) The flow chamber was constructed by two microslides with a smaller one being inserted into a bigger one, and two 45° mirrors coated with a high-reflected layer placed on each side of the chamber. The light path for a top-view (lower) and a side-view (upper) is illustrated [58]. B) μPIV components include a double-pulse Nd: YAG laser, a camera, a synchronizer, an amplifier and other optical components, as well as a microscope with fluorescent cubes and an objective lens. It also shows an optical path for a side-view μPIV imaging.

Two custom 45 degree mirrors with highly reflective surface coatings (Red Optronics; Mountain View, CA) were placed close to both sides of the chamber as shown in Figure 1A, where the basic principle of side-view observation is illustrated. When bright light is used, the light pathway for the side-view is LP1; while for the top-view, it is LP2.

The side-view μPIV set-up is shown in Figure 1B, as first successfully developed by Leyton-Mange *et al.* in 2006 [39]. A double pulse Nd: YAG laser (TSI; Shoreview, MN) with a wavelength of 540 nm was used as an illumination source. A PIVCAM 14–10 camera (TSI; Shoreview, MN) with a maximum capture speed of 5 fps was used to record PIV images. A synchronizer (TSI; Shoreview, MN) was connected to the camera and laser to control the time interval between successive frames.

### μPIV experimental procedure

Orange fluorescent microspheres (1.0 µm diameter) with an excitation/emission wavelength of 540/560 nm (Invitrogen; Carlsbad, CA) were chosen as tracer particles. The particles (1×10^10^ particles/ml) were diluted to 0.02% (by volume) in DMEM/F12 containing 1% BSA and incubated at 37°C for 24 hours in order to prevent possible aggregation among particles and non-specific binding between particles and cells before or during the experiment.

The flow was driven by a syringe pump (Harvard Apparatus, Holliston, MA) and unperturbed wall shear stress was calculated according to Equation 1
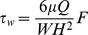
(1)where, μ is fluid molecular viscosity; Q is volume flow rate; W is the width of chamber; H is the height of chamber; and F is the correction factor for a rectangular channel with a finite aspect ratio. F is equal to 1.45 when an aspect ratio is 1.27 in this paper [58]. Two volumetric flow rates were chosen for this study, which are 73 µl min^−1^ and 365 µl min^−1^, corresponding to wall shear rates of 50 s^−1^ and 250 s^−1^ respectively. The viscosity of this solution was approximately 1.5 cP at room temperature, which was measured by a RotoViscoI cone-plate viscometer (ThermoMC; Madison, WI).

Lu1205 melanoma cells were mixed with N-formyl-methionyl-leucyl-phenylalanine (fMLP. Sigma; St Louis, MO) stimulated-PMNs at a 1∶1 ratio before the experiment. The cell suspension was first perfused into the flow chamber at a shear rate of 10 s^−1^ and then flow was stopped to let the cells settle and adhere to the Fibronectin coated surface. After 5∼10 minutes, experimental flow rates were then re-applied for at least 30 seconds to let adhered cells to reach equilibrium shapes and relative positions before images were captured.

**Figure 2:**
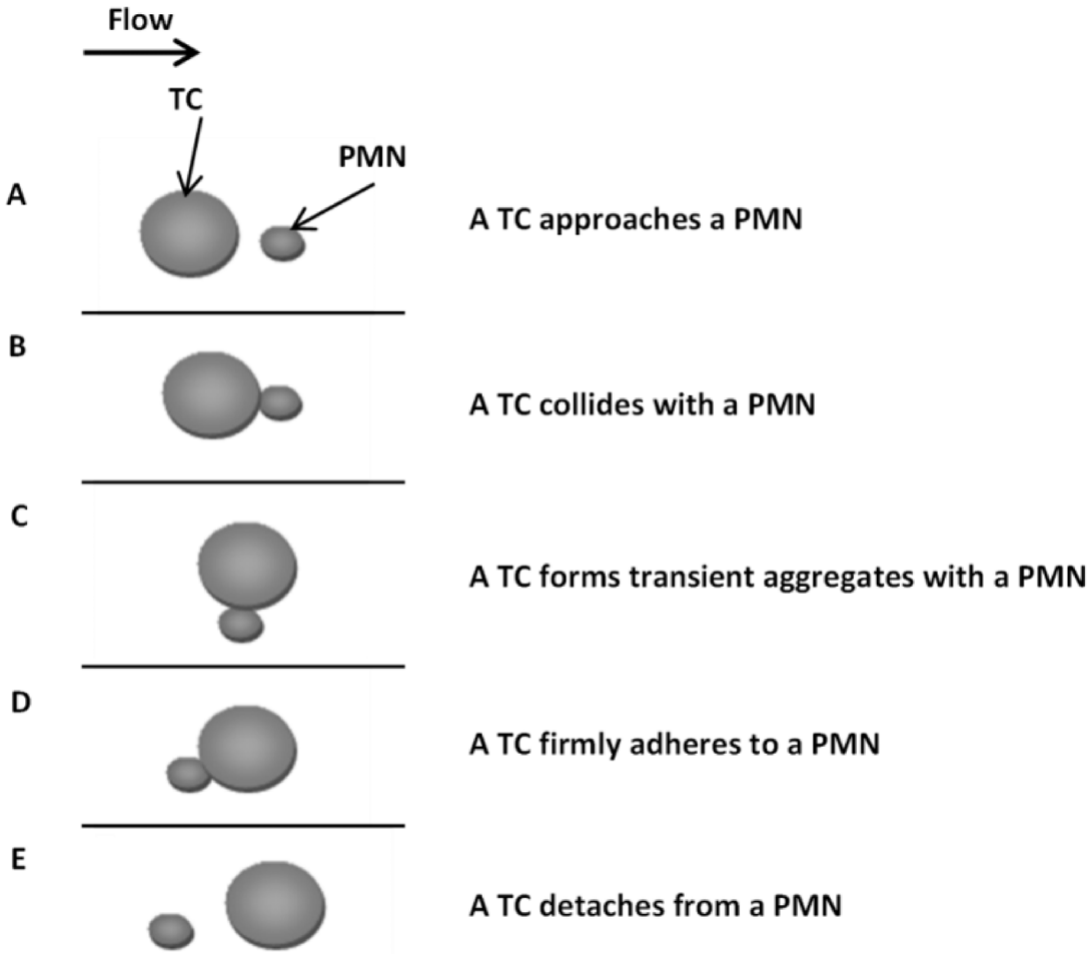
Relative positions of a TC with respect to an adherent PMN from a top-view. Five PMN-to-TC position states, A) a TC approaches a PMN; B) a TC collides with a PMN; C) a TC forms a transient aggregate with a PMN; D) a TC firmly adheres to a PMN; and E) a TC detaches from a PMN with the flow from left to right.

An important characteristic of Stokes flow is its quasi-steady state nature, i.e., the flow field at any instant in time is related only to boundary conditions at these time points, independent of the movement history of particles and other boundaries. When a free melanoma cell moves toward a firmly adherent PMN on the EC, the cells collide and form transient shear-resistant aggregates that facilitate TC arrest on the EC. Since this is a very low Reynolds number Stokes flow, relative PMN-to-TC positions were treated as five separate quasi-steady states (shown in Figure 2), where the velocity profile was assumed unchanged during the time when data were acquired (Δt between two frames). For each state, bright images of adhered cells were captured under top-view and side-view orientations, and 500∼1000 groups of μPIV images of the same field of view were acquired under side-view orientations then. Time intervals between two frames were set to 200 µs and 500 µs for low and high shear, respectively. Only the images in the middle 1/3 of the channel width were captured to avoid wall-edge effects [58].

### Data analysis

INSIGHT IV (TSI; Shoreview, MN), ImageJ (NIH) and NI-IMAQ (National Instruments; Austin, TX) were used for image processing. Average background images were generated by INSIGHT IV for frame A and frame B respectively, and then subtracted from raw images [29]. As there were not enough particles in one single image, ImageJ was introduced to overlap a stack of images together to increase the number of particles in a single image. Median and math min filters were also applied to increase the signal-to-noise ratios before overlap was performed depending to the quality of raw images [59], [60]. 50 images were overlapped to form a combined image in this study, and then NI-IMAQ was applied to add the missing dynamic boundary arising from the ImageJ process.

**Figure 3:**
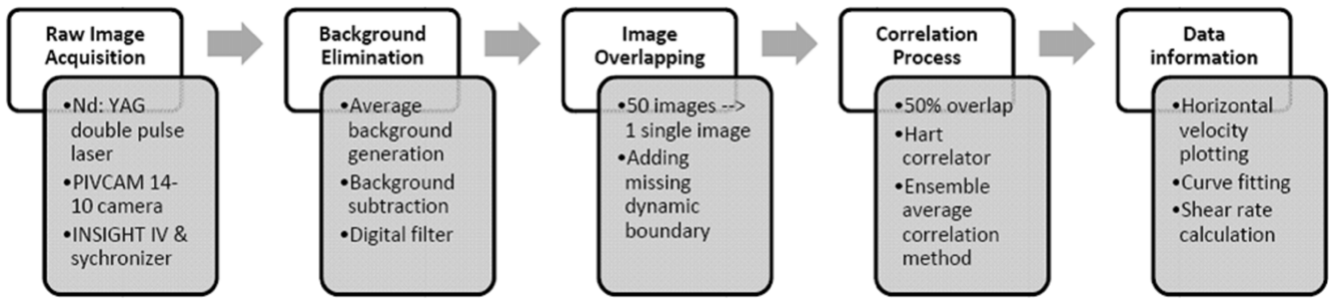
A procedure chart of the data acquisition and analysis for side-view μPIV experiments.

Processed images were imported into INSIGHT IV, where the image field was then gridded into a set of interrogation windows with a 50% overlap to satisfy Nyquist sampling. A Hart correlator was used to process the data. The ensemble average correlation method was chosen as it can significantly improve results compared with the average image method or the average velocity method [61]. Interrogation window sizes were 48×16 pixels for side-views and 32×32 pixels for top-views, under the 40× objective lens, and 64×64 pixels for the whole chamber under the 10× objective lens. The pixel sizes were estimated to be 0.16125 µm pixel^−1^ and 0.645 µm pixel^−1^ under the 40× and the 10× objective lenses, respectively. The process chart (Figure 3) shows the whole procedure starting from data acquisition through data analysis.

A single upstream horizontal velocity profile extending from the bottom of the chamber to 55 µm height (1/10 of the chamber height) was selected, and a linear regression curve was applied, yielding the slope of the near-wall velocity profile, in turn providing the wall shear rate. Another single velocity profile extending from the highest point of an adherent cell (velocity at this point was set to zero) up to two-cell heights was chosen, and a quadratic regression curve was fit, yielding the slope at the highest point of the adherent cell and a local shear rate there.

The calculation for bulk-flow Reynolds number in the side-view chamber and cell Reynolds number around an adherent PMN or TC are expressed by the following equations,
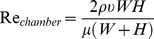
(2)

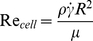
(3)where ρ is the density of flow; μ is the viscosity of flow; 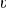 is average velocity in the chamber; W is the width of the chamber; H is the height of the chamber; 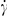 is the local shear rate of cell, and R is the cell radius.

### CFD simulations

As cell Reynolds number is very low in the near-wall region (The bulk Reynolds numbers associated with the low and high shear cases were 1.3, and 6.5 respectively), inertial effects are negligible compared to viscous effects and the flow can be considered as a Stokes flow. For incompressible Newtonian Stokes flow, the governing equations are
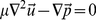
(4)

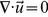
(5)


Carlos Rosales Fernández developed a program called stkSolver (http://software.ihpc.a-star.edu.sg/projects/stkSolver/stkSolver.php) under GPL License, which is a CFD solver for 3D Stokes flow based on the Boundary Element Method. The original version of stkSolver 1.0 was modified for our two cell system to be presented in this paper.

A 3-D model of a microchannel was generated based on the dimension of a side-view flow chamber, which is 700 µm wide, 500 µm high and 1000 µm long. Two model cells were placed on the bottom substrate in the center of the flow channel to correspond closely to experimental images. Pre-deformed cell shapes were generated by applying an analytical deformation equation [62],
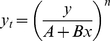
(6)where x and y are coordinates of non-deformed (spherical) cells; y_t_ is the transformed y-coordinate of a deformed cell; A, B and n are constants which are various for each deformed cell. Prior to transformation, the model cell dimension was scaled to match cell side-view images. Since it can be assumed that deformed adherent cells maintain their width dimension [58], z-coordinates were kept constant. Although there are some differences between the experimental images and the simulation images, they are comparable in terms of cell height and cell length [39]. A zero velocity condition was applied to all four surfaces of the chamber and along the outer surfaces of all cells. The analytical solution for a fully developed laminar velocity profile in a rectangular duct was used to specify the boundary conditions at the inlet and outlet, corresponding to the experimental volumetric flow rates of 73 µl min^−1^ and 365 µl min^−1^. A PYTHON script was written by the first author for chamber and cell mesh integration, cell dimension transformation, input files generation of stkSolver and stkSolver operation. Ensight 9.0 (CEI, Inc; Apex, NC) was used for visualization post-processing.

The stream wise drag force on the cell is computed as,
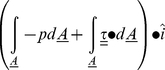
(7)where the surface integral is computed discretely by stkSolver by summing the pressure and viscous stress terms over each of the discrete surface elements of the adherent cell [63].

To validate the simulation technique, shear rates were calculated upstream and above the cell using the same regression methods as described for the μPIV data. The cases were chosen from previous side-view μPIV experiments performed by Leyton-Mange [39].

## Results

### Validation of the velocity profile in a side-view flow chamber using μPIV

**Figure 4:**
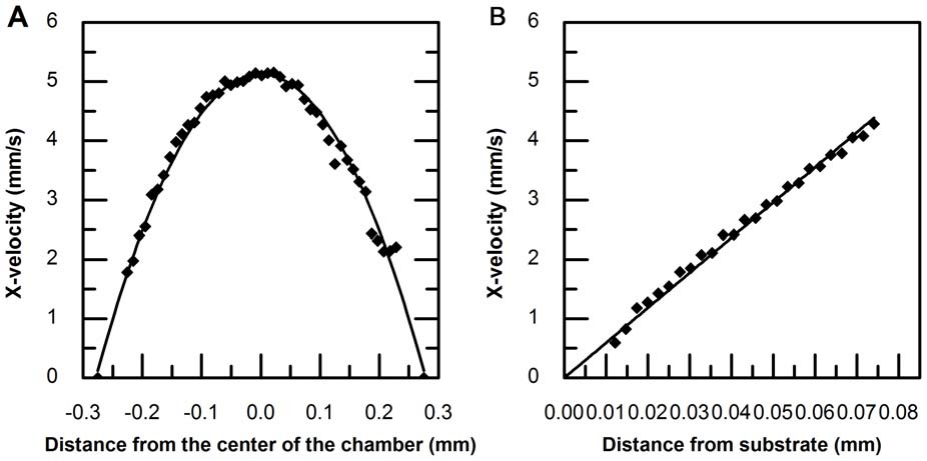
Velocity profile in side-view flow chamber measured by μPIV. The dimension for a side-view flow chamber is 550 µm high and 700 µm wide. Images were captured only in the middle 1/3 region across the width. A) X-velocity calculated from images taken by a 10× objective lens was plotted with respect to distance from the center of chamber showing a parabolic curve pattern. B) X-velocity calculated from images taken by a 40× objective lens was plotted with respect to distance from substrate and showed a linear curve pattern.

In order to validate the predicted velocity profiles for the chamber, side-view μPIV images were obtained using a 10× objective lens and analyzed using INSIGHT with a 64×64 pixel interrogation window size. The computed results exhibit a parabolic flow pattern that agrees closely with theoretical predictions (Figure 4A). Local velocity profiles were also calculated from images taken under a 40× objectives with an interrogation window size of 48×16 pixels, thereby establishing resolution requirements for characterizing the velocity profile in the near-wall region (Figure 4B).

### Velocity profile above two adherent cells from side-view μPIV and quadratic fitting

**Figure 5:**
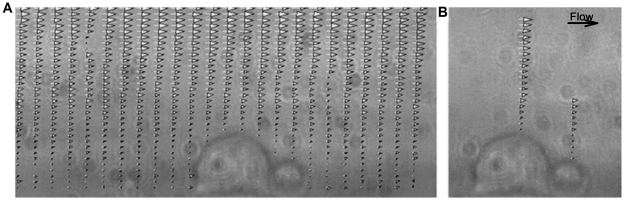
Experimental images showing velocity vectors around cells. Velocity profile obtained from the μPIV images were overlapped with the images of a TC (bigger one) and a PMN (smaller one) taken by bright field light source. A) Velocity profile around two adherent cells; B) two horizontal velocity profiles above the highest point of each cell surface.

**Figure 6:**
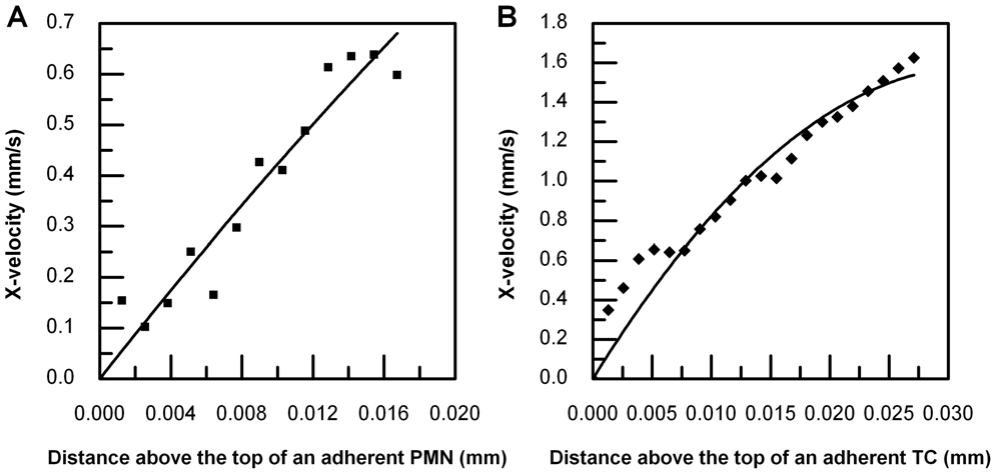
Shear rates above a TC and a PMN under a condition of low shear (73 µl min^−1^). A) Quadratic curve fitting for velocity profiles above an adherent PMN in 2-cell height region; B) Quadratic curve fitting for velocity profiles above an adherent TC in 2-cell height region.

The velocity profiles above two representative adjacent adherent cells (Figure 2B) were obtained, as shown in Figure 5A. Similar velocity profiles were also obtained for other PMN-to-TC position states from side-view μPIV experiments (data not shown). As described in the Methods section, a velocity profile above the highest point of an adherent cell was chosen (Figure 5B), which was plotted with respect to distance away from the cell surface and a quadratic curve was fitted to determine the local shear rate. As shown in Figure 6, the local shear rate above an adherent PMN is 61.21 s^−1^ with R^2^ 0.9095 and the local shear rate above an adherent TC is 97.221 s^−1^ with R^2^ 0.9351. All local shear rates in different PMN-to-TC position states at volumetric flow rates of 73 µl min^−1^ and 365 µl min^−1^ were calculated in the same way.

### Local shear rates above adherent cells in different relative positions

**Figure 7:**
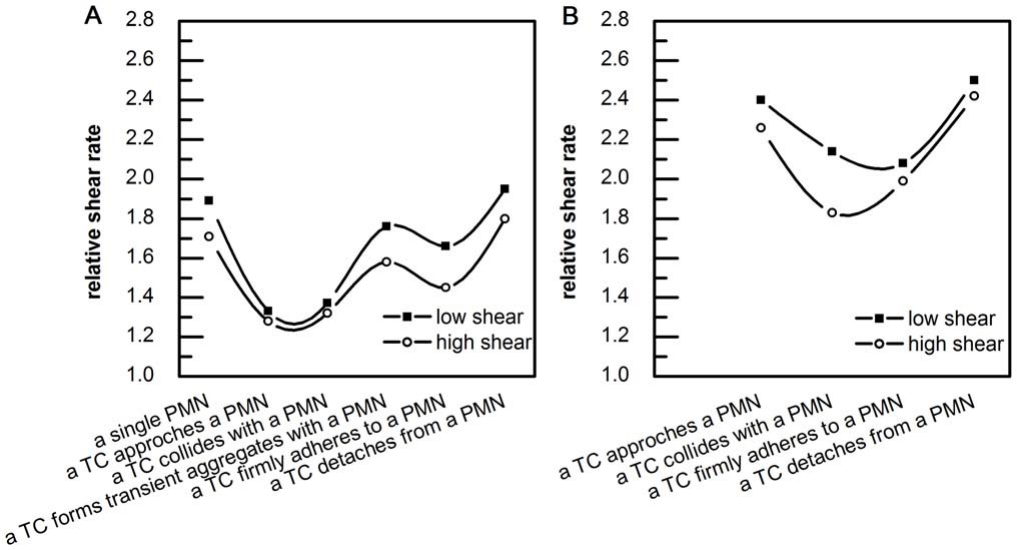
Relative Shear Rates above two adherent cells for different PMN-to-TC position states. A) Above an adherent PMN (including a single PMN without a TC); B) Above an adherent TC (no data for a transient aggregate state as the TC was not in the same plane as a PMN).

For varying PMN-to-TC position states (Table 1), the local shear rates above an adherent PMN are all lower than those above an adherent TC at low and high channel shear rates (50, 250 s^−1^ respectively), which suggests that TC disturbs the local flow patterns more than the PMN does. Results indicate that if a TC is moving toward a PMN that is considered to stay at a fixed location on the bottom of a chamber, the local shear rates above the adherent PMN would change when a TC is at different positions with respect to a PMN. In order to compare the local shear rate variation above the same type of cell under different shear conditions, the concept of Relative Shear Rate is introduced,
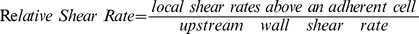
(8)Taking the relative shear rate above an adherent PMN at the state of “approach”, for example, the values for local shear rate and relative shear rate are 58.89 sec^−1^ and 58.89/44 = 1.338, respectively, under low shear conditions. If under high shear conditions, values for local shear rate and relative shear rate are 285.23 sec^−1^ and 285.23/222.3 = 1.283, respectively, which shows the relative shear rate is smaller in the case of high shear. The Relative Shear Rates above an adherent PMN and TC were plotted with respect to different PMN-to-TC position states at both low and high shear rates, respectively (Figure 7).

**Table 1:** Local shear rates above adherent cells calculated from μPIV results.

low shear					
upstream wall shear rate [s^−1^]	local shear rate [s^−1^]				
44.4	approach	collide	transient aggregates	adhere	detach
PMN	58.89	60.91	78.25	73.84	86.6
TC	106.72	95	n/a	92.17	111.1

high shear					
upstream wall shear rate [s^−1^]	local shear rate [s^−1^]				
222.3	approach	collide	transient aggregates	adhere	detach
PMN	285.23	294.41	351.44	321.64	401
TC	503.5	406.8	n/a	442.16	538.5

In Figure 7B, the curves show a biphasic trend. When a TC is relatively far away from an adherent PMN (>one cell diameter distance), the Relative Shear Rates above a TC were found to be above 2.2; when a TC interacts with an adherent PMN (e.g., a TC collides a PMN or a TC firmly adheres to a PMN), the Relative Shear Rates above TC were shown to be lower than 2.2, which might be due to the local flow disturbance associated with multiple cells at different PMN-to-TC position states. These results clearly illustrate that a reduced shear rate above a TC, which arises when it is approaches or is associated with an adherent PMN, enhances TC arrest on the EC. Figure 7A also shows a complicated trend that the lowest Relative Shear Rates above a PMN increases when a TC approaches to or collides with a PMN. More interestingly, the Relative Shear Rates above a PMN increases first as a TC forms a transient aggregate with a PMN, but then decreases slightly when a TC firmly adheres to a PMN; finally, it recovers to the magnitude above an isolated PMN. Similar pattern appear for low and high shear conditions. Moreover, results from Figure 7 show lower Relative Shear Rates above adherent cells under high shear conditions (with larger cell deformation), suggesting the important role of cell deformation in modifying the local hydrodynamic environment, and effect that in turn impacts cell-substrate adhesion.

**Figure 8:**
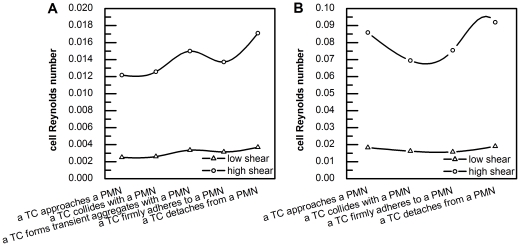
Cell Reynolds number calculations. A) an adherent PMN for five relative PMN-to-TC position states; and B) an adherent TC for four relative PMN-to-TC position states (the transient aggregate state was excluded). Open triangles (△) represent a low shear condition (73 µl min^−1^), and open circles (○) represent a high shear condition (365 µl min^−1^).

The bulk Reynolds number in the side-view chamber was 1.3 for the low shear case and 6.49 for the high shear case. Although the cell Reynolds numbers of both PMNs and TCs vary more significantly under high shear (365 µl min^−1^) than lower shear (73 µl min^−1^) conditions, the main trends of cell Reynolds numbers of the same type of cell are alike under different shear conditions (Figure 8). Generally speaking, cell Reynolds numbers of TCs are about 5 times of those of PMNs under the same flow condition (Table 2), which indicated that the drag coefficient (C_D_ = 24/Re) applied on TCs are about 1/5 of those applied on PMNs.

**Table 2:** Reynolds number calculated from μPIV results.

low shear					
bulk Reynolds number	cell Reynolds number				
1.29778	approach	collide	transient aggregates	adhere	detach
PMN	0.00251	0.00260	0.00334	0.00315	0.00369
TC	0.01821	0.01621	n/a	0.01573	0.01896

high shear					
bulk Reynolds number	cell Reynolds number				
6.48889	approach	collide	transient aggregates	adhere	detach
PMN	0.01217	0.01256	0.01499	0.01372	0.01711
TC	0.08593	0.06943	n/a	0.07546	0.09190

### Validation of a CFD model

**Figure 9:**
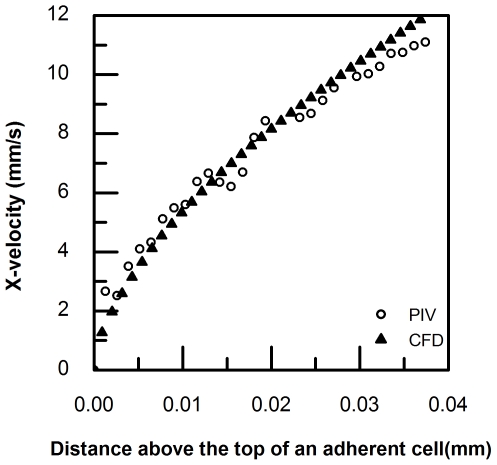
Comparison of μPIV measurements and CFD calculations. The X-velocity profile was plotted with respect to the distance away from the cell surface on the top. Open circle (○) and solid triangle (▴) represent μPIV data and CFD calculations under a condition of high shear (365 µl min^−1^), respectively.

In order to verify the CFD model used in this study, simulations were first conducted with pre-deformed cells in a flow chamber by applying a fully-developed velocity profile in a rectangular duct at the inlet and outlet of the chamber, as in our previous studies [39]. The calculation method followed that described earlier in the Methods section. The local shear rates and Relative Shear Rates above an adherent PMN and TC were compared between previous CFD and μPIV data [39] and the current data (Table 3). We find that the current CFD model agrees very well with the μPIV measurements for the upstream wall-shear rates under both low and high shear conditions. Local shear rates calculated above 1-cell height are much higher than at 2-cell heights, which indicate that the velocity gradient is much larger near the cell. Representative CFD results and μPIV data were plotted in Figure 9, showing that velocity profile curves above an adherent cell calculated using the current CFD model are comparable with those obtained from the current μPIV experiment.

**Table 3:** Comparison between current CFD and μPIV results with previous ones.

	Upstream wall shear rate	Shear rate in 2-cell height region		Shear rate in 1-cell height region	
		Local	Relative	Local	Relative
Previous CFD model	48 s^−1^	123.3 s^−1^	2.57	N/A	N/A
	232.8 s^−1^	507 s^−1^	1.98	N/A	N/A
**Current CFD model**	44.8 s^−1^	114.3 s^−1^	2.55	148.4 s^−1^	3.31
	224.3 s^−1^	519.3 s^−1^	2.32	694 s^−1^	3.1
Previous μPIV experiment	51 s^−1^	124.2 s^−1^	2.44	N/A	N/A
	257.7 s^−1^	502.4 s^−1^	1.95	N/A	N/A
**Current μPIV experiment**	44.4 s^−1^	N/A	N/A	N/A	N/A
	222.3 s^−1^	N/A	N/A	N/A	N/A

**Figure 10:**
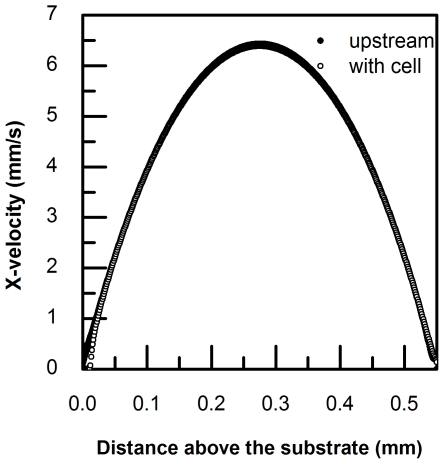
Upstream velocity profile (solid circle •) and velocity profile above a cell (open circle ○) were overlapped.

The velocity profiles of the chamber with or without an adherent PMN on the substrate calculated by CFD simulations were plotted together with respect to the distance above the substrate to visualize changes in horizontal velocity profiles. Except for the near wall region, (∼0.2∼0.3 mm high; Figure 10), there is no significant difference between the two curves, which demonstrates that an adherent cell only affects a local hydrodynamic environment within approximate 3-cell-height region above the substrate. As the data shows, a deformed cell would only affect a local hydrodynamic environment, which is in about 2-cell-height region above the highest point of the cell. However, the calculated Relative Shear Rates under both low and high shear conditions (Table 3) indicated that in a 1-cell-height region, effect of deformed cells on flow was much more dramatic, the value of which was 1.2∼1.4 times larger than that of 2-cell-height region. Moreover, it agrees well with the curve shape of velocity profiles above a single adherent cell, with a quadratic curve first followed by a linear curve [39].

### Velocity profiles above two adherent cells

**Figure 11:**
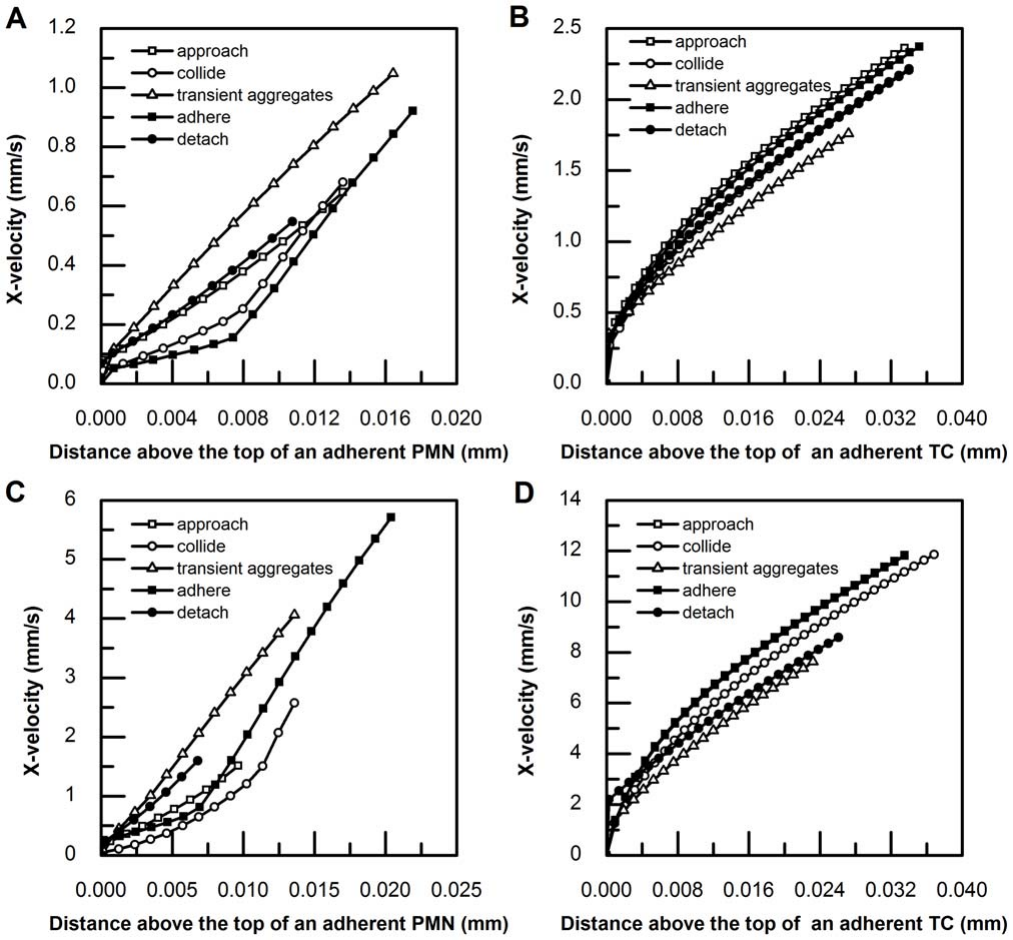
CFD simulations. X-velocity profile was plotted with respect to the distance away from the cell surface on the top, for all five relative PMN-to-TC position states, under both low shear (73 µl min^−1^) and high shear (365 µl min^−1^). A) Above an adherent PMN under low shear condition; B) Above an adherent TC under low shear condition; C) Above an adherent PMN under high shear condition; D) Above an adherent TC under high shear condition. Various symbols represent the position state of a TC approaching a PMN (□); a TC colliding with a PMN (○); a TC forming a transient aggregate with a PMN (△); a TC firmly adhering to a PMN (▪); and a TC detaching from a PMN (•), respectively.

A drawback of our current side-view μPIV technique is the resolution limitation associated with the working distance and magnification of the objective lens for image acquisition. CFD simulation provides a complementary approach for studying the details of the velocity profiles above multiple adherent and/or interacting cells, especially in the 1-cell height region near the cell wall where horizontal velocities have a larger gradient (Table 3). Thus, a number of CFD simulations were performed for cases of deformed cell shapes, relative PMN-to-TC positions, and flow conditions. 55 horizontal velocity vectors extending from the highest point of an adherent cell to 1/10 chamber high were calculated and plotted with respect to distance from the cell surface (Figure 11). In all cases, the velocity profiles above an adherent TC display a similar trend, a nearly quadratic curve immediately above the cell which transitions to a linear curve away from the cell surface. However, the horizontal velocity profile above an adherent PMN displays a different trend, especially immediately above the cell. Comparing the data for the same PMN-to-TC position state under various shear and high shear conditions, the observed profiles are very similar.

Although the velocity profiles above an adherent TC in all PMN-to-TC positions share a similar trend, the profile shapes above an adherent PMN are noteworthy. For example, when a TC approaches an adherent PMN, the velocity profile above the PMN looks like an exponential curve; when a TC collides an adherent PMN, the velocity profile above the PMN appears to fit an exponential curve but then changes to a linear curve, and this trend occurs faster under high shear rate conditions; when a TC forms a transient aggregate with an adherent PMN, the velocity profile above the PMN displays a linear curve; after a TC forms a more stable aggregate with an adherent PMN, the velocity profile above the PMN shows two linear segments with a smaller slope at the beginning, followed by a larger slope; lastly, when a TC detaches from an adherent PMN, the horizontal velocity profile above the PMN fits an exponential curve again.

### The recovery of velocity profiles above adherent cells

**Figure 12:**
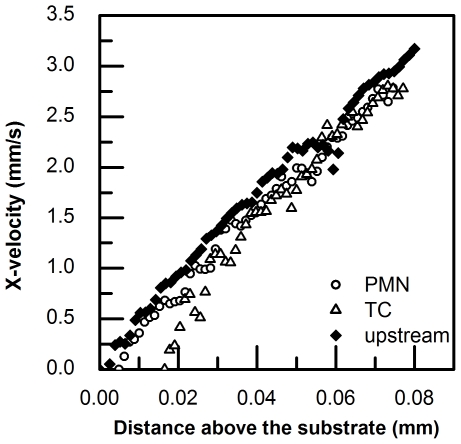
Velocity profile above an adherent PMN (open circle ○), above a TC (open triangle Δ) and the upstream (solid diamond ⧫).

In order to show how an adherent cell shape disturb the flow microenvironment, velocity profiles above adherent cells and in upstream, respectively calculated from μPIV data were plotted together with respect to the distance above the substrate. As Figure 12 shows, both the velocity profiles above an adherent PMN and an adherent TC are below those in upstream at the beginning and then become overlapped. The curve of velocity profiles above an adherent PMN recovers earlier than that of an adherent TC, showing that the flow is disturbed less by a PMN than a TC, which might due to the fact that the height of a deformed TC is larger than that of a deformed PMN under flow.

**Figure 13:**
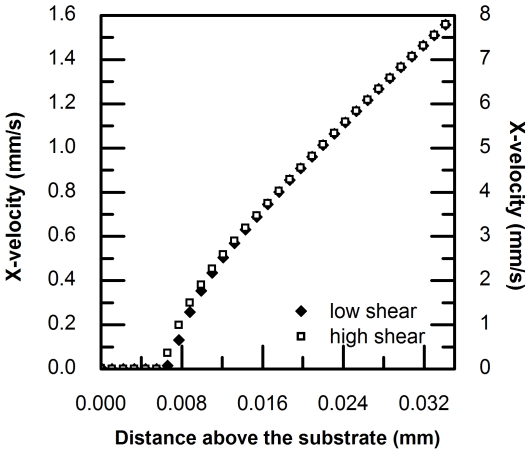
Comparison of velocity profiles above a single adherent PMN under low shear (73 µl/min; solid diamond ⧫) and high shear (365 µl min^−1^; open square □).

When a cell adheres to the substrate in flow, it deforms into a teardrop shape. It has been observed that under different flow conditions, the degree of cell deformation varies accordingly [58], [64]. Normally, for a firmly adhered cell, its length increases under a higher shear condition, while its height decreases. Figure 13 shows a comparison of computed velocity profiles above an adherent PMN under low and high shear conditions (71 and 365 µl ml^−1^, respectively), where cell height is 7.41 µm under low shear and 6.46 µm under high shear. The height of the adhered cell under higher shear condition was decreased by 12.8%.

### Drag forces calculated on deformed TCs

**Figure 14:**
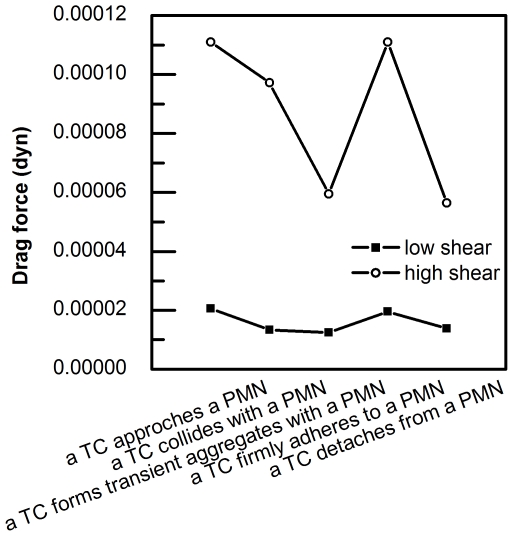
Drag forces calculated on deformed TCs under low shear (73 µl/min; solid square ▪) and high shear high shear (365 µl min^−1^; open circle ○).

Figure 14 illustrates drag forces calculated on deformed TCs with respect to all PMN-to-TC position states. The quasi-steady assumption invoked in the modeling is that the substrate and/or PMN adherent TC is in force equilibrium; adherent and contact forces equal and opposite to net flow force (pressure plus viscous drag). This assumption is valid for the nearly stationary substrate adherent configurations considered, and does not account for TC deceleration. When a TC is moving towards an adherent PMN and begins interacting with a PMN, the drag force on a TC decreases, especially under a high shear condition (365 µl ml^−1^), which helps a TC to form adhesive bonds with a PMN and the substrate.

## Discussion

Relative Shear Rates calculated from our side-view μPIV experiments were greater than 1, which agrees with the result that the peak shear rate is higher than the wall shear rate in the absence of a leukocyte reported by Pickard from their *in vivo* μPTV measurements. The increased magnitude of peak shear rates in our study was only around 50% of their results, and the differences might be explained by the observed focal plane, the near-wall plasma layer, non-Newtonian effects and the role red blood cell played in real blood flow. [65]. Chapman *et al.* also investigated the hemodynamic impact of leukocytes adherent to the wall of post-capillary vessels. Their results showed that there was a significant wall stress gradient generated by an adherent leukocyte if a cell-to-vessel diameter ratio is greater than 0.5 [66]. Comparing with the scale of cell-to-vessel diameter ratio (0.1–1.0) in their simulations, a cell diameter to chamber height ratio in our study was very small (0.015–0.029), but the local wall shear stress in the disturbed flow region did not equal the wall shear stress in undisturbed flow when the local shear rate rises.

Relative Shear Rates are not only affected by cell shape, but also influenced by the relative positions between the two cells. As seen in Figure 11B, under low shear rates, the velocity profiles above an adherent TC are not significantly affected by PMN-to-TC position states; whereas the velocity profiles above an adherent PMN vary considerably (Figure 11A) with position state. Referring to Figures 11C and D, at high shear rates, the TC exhibits more significant variation with position state, but still is less sensitive to state than the PMN. These results indicate that adherent PMNs are generally more affected by PMN-to-TC position states. This is due to the much larger size of the TC (∼16 µm), compared to the PMN (∼8 µm), the local mean flow being more significantly disturbed by the larger blockage – this effect dominating the more subtle effects of relative position state.

When cells adhere to the vascular wall, shear above the cells become larger than that in the upstream due to mean flow blockage and attendant accelerations around the top of the cell. In previous cell adhesion studies, Reynolds numbers based on upstream flow shear rates were used to parameterize cell drag coefficients [21], [25], [37]. The present results indicate that these drag coefficients are overestimated because local cell Reynolds numbers are larger than those based on undisturbed shear (and thereby C_D_ is lower), especially for larger cells with less deformation.

TCs cannot directly adhere to ECs in blood flow without significant adhesion to other cells such as PMNs [16], [24]. One possible reason why adherent PMNs would facilitate TCs adhesion to ECs is cell contact time, which is inversely related to the shear rate. When a TC interacts with an adherent PMN, local shear rates decrease (Figure 7B) resulting in increased contact time, in turn leading to the formation of a larger number of adhesive bonds. Another reason is that drag forces on TCs also decrease as shown in Figure 14. In general, an increase in drag force implies an increase in cell adhesion (due to cell deformation and cell-substrate contact area changes) when a single cell is considered. However, when we focused on a two-cell system, the proximity of the PMN to the TC serves to modify the drag force acting on the TC. For example, Chapman and Cokelet reported that if two spheres are aligned on the same side, the drag force on each sphere is less than that on an isolated sphere, and the drag force would reach minimum when the two spheres are touching one another [67]. Similarly, we found that when TC forms transient aggregates with an adherent PMN, the drag force decreases to promote TC become firmly captured by PMN. Therefore, apparent TC arrest in the downstream of an adherent PMN may be a result of both transient contact time and instantaneous drag force.

A number of experimental and computational of results have been presented for TC-PMN systems in this paper. We have demonstrated that both cell deformation and cells' relative positions contributed to the disturbance of local hydrodynamic environment, which may facilitate TC's capture on EC in blood flow. However, there are other factors besides cell deformation, such as microvillus deformability and receptor-ligand biding kinetics, affecting the behavior of cells besides cell deformation. The interplay of these factors has been modeled together to reproduce cell rolling over a ligand-coated surface [55]. Our future work will focus on several potential improvements to these studies. First, the experiment could be performed under more physiological conditions, for example, endothelium cells monolayer could be used to replace Fibronectin for the substrate. Second, improved camera frame-capture speeds will decrease the scatter when quasi-steady conditions are considered. Third, the microscope system could be modified to capture both side-view and top-view images simultaneously. Lastly, the, CFD model could be coupled with biochemistry and kinetics modeling [55], [56], [62] for more accurate simulations of the cell interaction process including critical physics such as contact area evolution and interactional contact time.
